# Role of Artificial Intelligence in Health Research: Opportunities, Challenges, and Implications for Medical Education

**DOI:** 10.7759/cureus.106579

**Published:** 2026-04-07

**Authors:** Raywat Deonandan

**Affiliations:** 1 Health Sciences, University of Ottawa, Ottawa, CAN

**Keywords:** algorithmic bias, artificial intelligence, clinical diagnosis, drug discovery, health research, machine learning, medical education

## Abstract

Artificial intelligence (AI) is fundamentally reshaping the landscape of health research, with applications spanning drug discovery, clinical diagnosis, patient monitoring, and medical education. This paper examines the transformative potential of AI technologies, including machine learning, deep learning, and natural language processing, across these domains, while critically evaluating the ethical, legal, and infrastructural challenges their adoption entails. Particular attention is paid to issues of algorithmic bias, data privacy, clinical accountability, and the growing imperative to integrate AI literacy into medical training. Particularly relevant to the Canadian context, this paper contends that realizing AI's promise in health research demands simultaneous progress on three fronts: technical innovation, a contextually grounded ethical framework, and a healthcare workforce equipped to navigate an AI-augmented practice environment.

## Editorial

Introduction

Artificial intelligence (AI) is rapidly transforming the landscape of health research, offering unprecedented tools and methodologies that promise to enhance the efficiency, accuracy, and comprehensiveness of scientific investigations. The integration of AI technologies, such as machine learning algorithms, natural language processing, and deep learning frameworks, into health research has facilitated the analysis of vast amounts of data, ranging from genetic sequences to electronic health records and real-time biometric monitoring. This fusion not only accelerates discovery but also enables more personalized approaches to medicine, potentially revolutionizing patient care and public health strategies.

However, the adoption of AI in health research is not without challenges. Issues related to data privacy, algorithmic bias, model performance variability, and the risk of clinician overreliance are at the forefront of ongoing debates. This paper argues that the Canadian context, characterized by chronic physician shortages, an aging population, provincial variation in health data governance, and a publicly funded system under sustained pressure, creates both distinctive urgencies and distinctive vulnerabilities that have received insufficient attention in a literature dominated by American and European perspectives. Rather than treating AI's clinical applications, ethical implications, and educational dimensions as separate domains, this paper argues that they are interconnected. How AI tools are deployed in practice shapes the ethical problems that arise and determines how clinicians, educators, policymakers, and institutions must respond.

Drug discovery and clinical applications

One of the more exciting advancements promised by machine learning is its potential for accelerating drug discovery. One of the primary concerns associated with drug design and development is time consumption and production cost. Overall process inefficiency, inaccurate target delivery, and inappropriate dosing all inhibit the pace of pharmaceutical development [1]. Predictive neural networks are already being trialed to monitor drug release parameters, assist in biomarker identification, scale up pharmaceutical manufacturing, measure bioactivity, identify physicochemical properties, predict toxicity, and identify mode of action [1].

While it is unlikely that a generative model could automatically produce drug candidates with fully optimal properties [2], the inclusion of AI in drug discovery pipelines can screen millions of compounds in a matter of hours, identifying potential candidates that would have taken years to find using traditional methods [3]. This represents a paradigm shift in pharmaceutical research, with AI systems demonstrating particular promise in the development of treatments for complex conditions such as neurodegenerative diseases, where traditional compound screening has historically been slow and resource-intensive [4].

Beyond the laboratory, AI is increasingly being deployed to support clinical decision-making. Some algorithmic approaches have achieved diagnostic accuracies comparable to those of human specialists across several specializations, including radiology, dermatology, and ophthalmology [5]. In contexts where specialist access is limited (such as in rural or under-resourced healthcare settings), AI-assisted diagnostics can serve as something of an equalizer, providing high-quality decision support where such would otherwise be unavailable [6].

AI tools might also reduce the administrative burden on clinicians. Natural language processing systems capable of transcribing clinical notes, drafting referral letters, and generating discharge summaries can free physicians from tasks that, while necessary, do not require clinical expertise. In Canada, where the healthcare system is under sustained pressure from physician shortages, an aging population, and elevated emergency department wait times, tools of this kind could meaningfully improve the system’s capacity to meet patient demand [7].

Big data in health research

A central advantage of AI in health research is its ability to extract meaningful patterns from datasets of a scale and complexity that are far beyond human analytical capacity. Electronic health records, genomic databases, medical imaging archives, and real-time wearable device outputs collectively generate enormous quantities of data, both structured and unstructured. AI systems, and particularly deep learning architectures, are uniquely well-suited to mining these resources for clinically actionable insights [8].

In the context of population health research, AI enables the identification of risk factors and disease trajectories across large cohorts with a speed and granularity that traditional epidemiological methods cannot match. Predictive models trained on electronic health record data have demonstrated the ability to anticipate adverse clinical events, such as sepsis, deterioration in hospital inpatients, and readmission following discharge, with sufficient lead time to enable preventive intervention [9]. These capabilities represent a meaningful shift from reactive to proactive healthcare, with significant implications for both clinical practice and health system efficiency.

The convergence of AI with genomics has similarly opened new frontiers. Machine learning models trained on large-scale genomic and proteomic datasets have accelerated the identification of disease-associated variants and potential therapeutic targets, supporting a more precise and individualized approach to medicine [10]. These developments identify AI as not merely a tool for existing research workflows, but as an enabling technology that makes entire categories of investigation newly feasible.

Challenges and ethical considerations

Despite the considerable benefits outlined above, the deployment of AI in healthcare raises serious ethical, legal, and practical concerns that must be addressed alongside its promise if the technology is to be used responsibly. Crucially, the same capabilities that make AI systems appealing in clinical contexts (their speed, scale, and apparent precision) also introduce risks that a balanced appraisal cannot overlook.

Chief among these is the issue of algorithmic bias. AI systems trained on historical datasets risk encoding and amplifying the biases in those datasets, producing models that perform well for some demographic groups while systematically underserving others. Studies have documented disparities in the performance of diagnostic AI across racial, gender, and socioeconomic categories. If left unaddressed, these disparities could deepen existing health inequities rather than reduce them [11].

Closely related is the problem of model performance variability, which has received insufficient attention in optimistic accounts of AI in healthcare. Accuracy levels reported in controlled research settings often do not carry over to real-world clinical environments, where patient populations, data quality, imaging equipment, and documentation practices differ from those used in training [11,12]. A diagnostic model validated on data from a large urban academic hospital might perform less reliably when used in a rural setting. Yet, this is precisely the context where AI has been most enthusiastically promoted as an equalizer. This performance gap is not merely a technical inconvenience. Rather, in high-stakes clinical decisions, the gap can compromise patient safety. Rigorous, context-specific validation prior to deployment must therefore be treated as an ethical requirement, not an optional step.

The risk of clinician overreliance on AI outputs compounds these concerns. When AI systems are integrated into diagnostic or decision-support workflows, there is evidence that some clinicians may defer to algorithmic recommendations even when those recommendations are incorrect; this phenomenon is sometimes termed automation bias [13]. This is pronounced when AI outputs are presented with high confidence, or when time pressures discourage independent verification. Overreliance is not simply a matter of individual clinical judgment. Rather, it reflects systemic design choices about how AI tools are presented, what override mechanisms are in place, and how healthcare professionals are trained to engage critically with AI outputs. AI should be positioned as a decision-support tool that augments clinical reasoning rather than supplants it, and institutional protocols must explicitly reflect this distinction.

Data privacy presents another significant challenge. The effective training of AI models in healthcare requires access to large volumes of sensitive patient information. Ensuring that this data is collected, stored, and used in compliance with applicable privacy legislation and with patients' informed consent is both ethically necessary and operationally complex. The aggregation of data across institutions and jurisdictions, while often technically desirable, introduces additional legal and governance challenges that vary considerably between regulatory environments [12].

The question of accountability is equally pressing. When an AI-assisted clinical decision leads to patient harm, the distribution of responsibility among developers, institutions, and individual clinicians is not always clear under existing legal frameworks. This ambiguity could create a diffusion of accountability that ultimately undermines trust in both AI systems and the healthcare institutions that deploy them [13]. Regulatory bodies in many jurisdictions are still in the early stages of developing frameworks adequate to these challenges, and the pace of AI adoption in clinical settings risks outrunning the governance structures needed to ensure it is safe.

Finally, there are legitimate concerns about the effect of AI on the relational dimensions of patient care. Some patients will prefer receiving care from a clinician who can offer empathy, so-called lived experience, and emotional understanding in ways that AI systems, however sophisticated, cannot replicate. The risk that widespread AI adoption could erode this quality of care should inform how and where these technologies are deployed [5]. Additionally, certain roles in the healthcare workforce (such as medical scribes, administrative staff, and some categories of diagnostic technicians) may face displacement as AI takes on tasks currently performed by humans, raising important questions about workforce planning and the broader social ethics of automation.

AI and medical education

Given the rapidly expanding role of AI in clinical practice and research, there is a growing consensus that medical education must evolve to equip future healthcare professionals with the knowledge and skills necessary to work effectively alongside these technologies. Physicians who lack a basic understanding of how AI models are trained, their limitations, and how to critically evaluate AI-generated recommendations are poorly positioned to use them safely or to advocate effectively for their patients. This is particularly concerning when algorithms produce erroneous outputs such as "hallucinations" [14].

Medical curricula at both the undergraduate and postgraduate levels are beginning to incorporate AI-related content, though the degree of integration and the pedagogical approaches adopted vary widely across institutions [15]. Generative AI tools, such as large language models, are increasingly being used by medical students for learning support, case preparation, and self-assessment, creating both opportunities for enhanced learning and risks associated with overreliance on outputs that may be inaccurate or incomplete [16].

The emerging view in the literature is that AI literacy should not be added as a standalone module but woven into training across clinical domains [5]. To operationalize this, the curriculum should be structured around several core competency areas. First, foundational AI literacy should ensure that trainees understand the basic principles of how machine learning models are trained, validated, and updated. This should include key concepts like training data, overfitting, and generalizability. This need not require deep technical expertise but should produce graduates capable of critically appraising AI tools rather than treating them as black boxes. Second, clinical application and output interpretation should be taught within domain-specific contexts. For example, radiology trainees can work with AI-assisted image analysis. Or internal medicine residents can engage with AI-generated risk stratification outputs. This allows students to learn to interrogate algorithmic recommendations against their own clinical reasoning rather than defaulting to the AI. Third, students should be trained to identify automation bias in themselves and their colleagues, to recognize when model outputs are likely to be unreliable due to patient population mismatch or data quality issues, and to understand the circumstances under which AI tools have historically underperformed for specific demographic groups [11].

Beyond technical competencies, trainees require structured education in the ethical and professional dimensions of AI in healthcare. This should include dedicated instruction in algorithmic bias and health equity, privacy and data governance frameworks, and the legal implications of AI-assisted decision-making, including the accountability questions raised when AI outputs contribute to adverse patient outcomes [13]. Case-based teaching formats are particularly well suited to this content, as they allow students to reason through realistic scenarios in which clinical, ethical, and technical considerations intersect.

Finally, the curriculum should address the appropriate use of AI tools in learning itself. Given that medical students are already using large language models for study support and case preparation [16], educational institutions have a responsibility to develop explicit guidance on critically evaluating AI-generated content, verifying outputs against authoritative sources, and understanding the academic integrity implications of AI-assisted work. Embedding this guidance early in training will better prepare graduates for the professional norms they will encounter in clinical practice. Taken together, these competency areas point toward a model of AI education that treats AI fluency not as a supplementary technical skill but as a core dimension of contemporary clinical professionalism.

Conclusion

AI holds extraordinary promise for health research and clinical practice. Its ability to accelerate drug discovery, enhance diagnostic accuracy, analyze population-scale datasets, and reduce the administrative burden on clinicians positions it as one of the most consequential technological developments in the history of medicine. However, these capabilities must be harnessed with care. Algorithmic bias, data privacy, accountability gaps, and the risk of eroding the human dimensions of care are real concerns that demand sustained attention from researchers, clinicians, ethicists, and policymakers alike.

Realizing the full potential of AI in health research will require more than technical progress. It will require the development of robust governance frameworks, the cultivation of diverse and representative training datasets, and the education of a healthcare workforce that is both technically literate and ethically grounded. Interdisciplinary collaborations between such domains as computer science, medicine, law, and the social sciences will be essential to ensuring that these technologies serve the interests of all patients equitably and transparently.
